# Performance of Three Multipurpose Disinfecting Solutions with a Silicone Hydrogel Contact Lens

**DOI:** 10.1155/2015/216932

**Published:** 2015-03-31

**Authors:** Nery García-Porta, Laura Rico-del-Viejo, Helena Ferreira-Neves, Sofia C. Peixoto-de-Matos, Antonio Queirós, José M. González-Méijome

**Affiliations:** ^1^Clinical & Experimental Optometry Research Lab, Center of Physics (Optometry), University of Minho, 4710-057 Braga, Portugal; ^2^Ocular Surface and Contact Lenses Research Group, University of Santiago de Compostela, 15782 A Coruña, Spain

## Abstract

*Purpose*. To evaluate the clinical performance of a silicone hydrogel (Si-Hy) soft contact lens (CL) in combination with three different multipurpose disinfecting solutions (MPDSs). *Methods*. This was a prospective, randomized, single-masked, crossover, and comparative study in which 31 habitual soft CL wearers were randomly assigned to one of the three MPDSs (Synergi, COMPLETE RevitaLens, and OPTI-FREE PureMoist) for 1 month with a 1-week wash-out period between each exposure. All subjects were successfully refitted with a Si-Hy CL (Biofinity). Subjects were then scheduled for follow-up visits after 1 month of lens wear, being evaluated at 2 and 8 hours after lens insertion. Visual Analogue Scales (VAS) were used to gauge comfort rating. *Results*. The tarsal conjunctiva showed a significantly different degree of lid redness between the MPDSs at the 2-hour visit (*P* < 0.05, Kruskal-Wallis test), being lower for COMPLETE RevitaLens compared to the other two MPDSs (Mann-Whitney *U* test). Furthermore, a significantly different degree of lid roughness at the 8-hour visit was seen (*P* < 0.05, Kruskal-Wallis test), being higher for Synergi (Mann-Whitney *U* test). The subjective comfort was similar with the three MPDSs. *Conclusion.* Tarsal conjunctival response should be also considered in the context of the clinical performance of MPDs at the ocular surface.

## 1. Introduction

Soft contact lens (CL) wearers frequently report symptoms of discomfort and dryness [1–3]. Despite that, soft CLs continue accounting for about 90 percent of all CLs fitted and silicone hydrogel (Si-Hy) CL in daily wear basis is the modality of CLs most common fitted [4]. Although soft CLs have changed significantly during the last years with the introduction of new materials, designs, and care systems, discomfort continues being the most frequent reason for CL wear discontinuation [5–8]. The precise etiology of these symptoms is unknown, but it is clear that it is multifactorial. The comfort depends, among other things, on several factors related to the CLs, but also it is related to the tear quality of the CL wearers and with lens care regimen used [9–11].

It has been suggested that a lower dehydration rate of CLs might be an important factor to reduce dryness symptoms [12]. González-Méijome et al. have seen that worn Si-Hy CLs have lower ability to retain bulk hydration compared to unworn Si-Hy CLs [13]. In this regard, avoiding on-eye dehydration of soft CLs is one of the challenges for ophthalmic materials scientists and CLs industry. Moreover, a more stable tear film at the lens surface will contribute positively to the optical quality of the eye [14, 15], thus potentially reducing patient symptoms.

Nowadays, the CLs care industry is committed to develop more effective care systems in the form of multipurpose disinfecting solutions (MPDSs) that attempt to improve the safety and efficacy of soft CLs. Their results are good from the antimicrobial and clinical performance perspectives [16–18]. In fact, The MPDSs are commonly used due to their simplicity and cost effectiveness, enhancing the compliance [19]. Nevertheless, after soaking the CLs in the MPDSs during the night, the liquid remains present in the CLs. So, when the CLs are placed on the eyes, the liquid is released in amounts that are dependent on the CL material chemistry [20–22]. In this regard, asymptomatic low-grade punctate corneal staining associated with toxicity of CLs care solutions is common [23–26]. It has been seen that the toxic staining is most likely to be observed within 2–4 hours after inserting the CL [27]. This kind of staining tends to be extensive but superficial and transient so, in most cases, it is clinically insignificant and does not require cessation of CL wear [27, 28]. However, some combination of MPDSs with some types of Si-Hy CLs can lead to toxic staining of sufficient severity to result in discontinuation of CL wear [28].

Currently, new products are being released to the market, some of them with the claim of including active ingredients to improve comfort. Other products include more complex formulations to strength disinfection activity. This is necessary to evaluate the effects on the comfort and physiological status of the ocular surface. Therefore, the purpose of this study was to evaluate and compare the clinical and subjective performance, as well as the dehydration rates of a Si-Hy CL in combination with three different MPDSs for sequential periods of 1 month separated by 1 week without CLs.

## 2. Methods

This was a prospective, randomized, single-masked, crossover, single center, and comparative study. All procedures conformed to the tenets of the Declaration of Helsinki. The Ethics Committee of the School of Sciences of the University of Minho (CEECUM) approved the study, and informed consent was obtained from each individual prior to the initiation of the study. Subjects must satisfy the following inclusion and exclusion criteria (Table 1) to be eligible for the study. To avoid differences related to the CL fit, the subjects that could need to use toric or multifocal CLs were excluded. Moreover, ageing is a risk factor to suffer dry eye and the use of some medication, such as antihistamines or antidepressants, may have influence over dryness symptoms. Therefore, only healthy young subjects with myopic refraction and astigmatism less than 1.0 D were included in the study. The age limitation was imposed to warrant uniformation and be consistent with the most frequent age range of contact lens wearers in most countries. In case the participants used to use artificial tears or rewetting drops without preservatives while they wore their habitual CLs, they were allowed to use them but always in the same way. This means, if they used to put a drop during the morning and another one during the afternoon, they should do the same during the 3 months of follow-up period, the same rewetting drops, and the same times per day with the three MPDSs. This will allow us to evaluate the patients in the same conditions they were used to and measure differences against baseline and between MPDSs under the same conditions.

All subjects underwent a complete ocular examination to ensure that each individual met all of the inclusion criteria of the study. After recruitment, 31 subjects were included in the study and they were asked to discontinue their CL wear for 1 week before attending the baseline visit. In the baseline visit, a new pair of CLs and a new MPDS were dispensed and then, the subjects were scheduled for follow-up visits after 1 month of CL wear, being evaluated at 2 and 8 hours after lens insertion. After the follow-up visits, subjects were instructed to use their spectacles for 1 week and were then rescheduled for dispensing a new MPDS and a new pair of CLs. This procedure was repeated for each MPDS, starting each subject by one MPDS according to a predetermined randomization schedule.

### 2.1. Contact Lens and MPDS Care Systems

All participants were successfully refitted with Biofinity (Comfilcon A 48% water content, Coopervision) Si-Hy CL and were instructed to wear the CLs in daily wear basis. This is a Si-Hy CL that is replaced monthly, the Si-Hy CLs are the most used nowadays and the majority of the CLs fitted in the South of Europe, the region where this study was performed, are replaced on a monthly basis [4]. Beyond this, Biofinity represents a last generation Si-Hy with high oxygen transmissibility and at the same time mid water content and low modulus. We believe that it is a good representative of the CL market.

The three MPDSs used in this study were Synergi (Sauflon, UK), COMPLETE RevitaLens (Abbott Medical Optics, Santa Ana, CA), and OPTI-FREE PureMoist (Alcon, Fort Worth, TX).  Table 2 shows the technical details of the solutions involved in the comparison. Each solution was used following the manufacturer's instructions and the lens case provided by the manufacturer of the MPDS, when possible. All products were used with a rub-and-rinse step. The subjects were not masked for lens care solutions but the clinician was masked for the solution that each participant was using during each month.

### 2.2. Tests Performed

The participants graded their ocular comfort experiences on a 1 to 10* Visual Analogue Scales (VAS)*. Therefore, VAS were used to gauge comfort ratings at baseline, with their habitual soft CLs, and then after 15 and 30 days using each MPDS. At the end of the study, the participants had to show the preference by one of the three MPDSs by forced choice. In this questionnaire, the participants had to say at what time they used to put and remove their CLs, as well as at what time they started to feel the CLs uncomfortable or felt dryness with the CLs, if these symptoms took place. Moreover, they were asked about the comfort and dryness with their CL with each MPDS. Their satisfaction with the cleanliness and with CL clear vision was also assessed. As there was not any programmed visit after 15 days using each MPDS, the participants were asked to answer the VAS questionnaire at home and to bring them at the 1-month visit.

The ocular surface was examined with* slit lamp biomicroscopy* evaluating corneal staining, conjunctival staining, and bulbar, limbal, and lid redness, as well as tarsal roughness. The scores were recorded according to the CCLRU grading scales [29]. This scale has four images for each condition that increase in severity from 1, which means “very slight,” to 4, which means “severe.”

The tear quality was evaluated with noninvasive tear break-up time* (NIBUT)* and with the tear break-up time* (BUT)* tests. NIBUT was measured using the Medmont topographer with and without CL. BUT was measured with the slit lamp biomicroscopy after instilling sodium fluorescein (NaFL) with a prepared strip of NaFL. A yellow barrier filter was used to enhance the contrast. Both tests were assessed after asking the subjects to blink a couple times and the measurements were performed three times to obtain a more reliable value.

The* CLs* were* weighed* with a digital balance (model AT210, Mettler Toledo, Giessen, Germany) at baseline, after opening the blister of each CL, and at 1-month follow-up visits, after 2 and 8 hours wearing the CL. At 1-month visits, immediately after lens removal, the lens was placed in a sterile holder and weighed. The analytical balance has a scale capable of measuring within 0.001 g. An experienced technician weighed the CL three times, after soaking the CL, and removed the excess of liquid. The measurements were performed always following the same procedure. Values obtained were recorded and compared against the values obtained before lens insertion for each given CL (baseline). Dehydration rates were derived according to previously described methodology by the equation: *Weight* *loss* = [(*Worn* *lens* *mass* − *Baseline* *mass*)/*Baseline* *mass*] × 100.

During the dispensing visits, the participants underwent slit lamp examination, evaluation of NIBUT with and without CL and BUT. These values were used as baseline values for each MPDS.

### 2.3. Statistical Analysis

Statistical analysis was conducted using SPSS v.19.0. Shapiro-Wilk test was used to evaluate normal distribution of data. Analysis of variance (ANOVA or Kruskal-Wallis test, depending on data distribution) with Bonferroni post hoc correction was used to compare the clinical and subjective outcomes of the three MPDSs to check for differences between solutions at a given visit or between follow-up visits for a given MPDS. Mann-Whitney *U* test was used to evaluate differences among the MPDSs in lid response at a given visit. Statistical significance was set at the level of *α* = 0.05. Sample size was estimated for an 80% statistical power to detect differences of 2 values in comfort ratings, 4 seconds difference in NIBUT over the lens or differences of 1.5% in dehydration rates considering a statistical significance value of 0.05. To avoid the duplication of the sample resulting from the interaction between both eyes from the same patient, only the left eye from each patient was used for statistical analysis.

## 3. Results

Of the 31 subjects enrolled, 25 were females (80.6%) and 6 were males (19.4%), with a mean age of 23 ± 4 years. Average spherical equivalent refraction was −2.75 ± 1.30 D for the right eye and −2.91 ± 1.31 D for the left eye.

There was only one discontinuation during the third month of the study because one subject felt disappointed after breaking 2 CLs with the case of Synergi solution. In this regard, it is interesting to mention that 7 CLs were broken during the study and 6 of them were broken while the subjects were using the Synergi solution with its case. Of course, all CLs were replaced and the appointments reprogramed to make the visit after 1 month of CL use with each MPDS.

### 3.1. Subjective Questionnaire

The three MPDSs performed similarly regarding the subjective perception of the subjects, not finding statistically significant differences among the three MPDSs for any question in the VAS questionnaire (*P* > 0.05, Kruskal-Wallis test).

The participants showed a consistent pattern of 12 to 13 hours/day of lens wear for 6 to 7 days/week. During this period, they reported 11 to 11.5 hours of comfortable wear over the month of lens wear. Differences between MPDSs regarding total time or hours of comfortable lens wear were not statistically significant.

**Figure 1:**
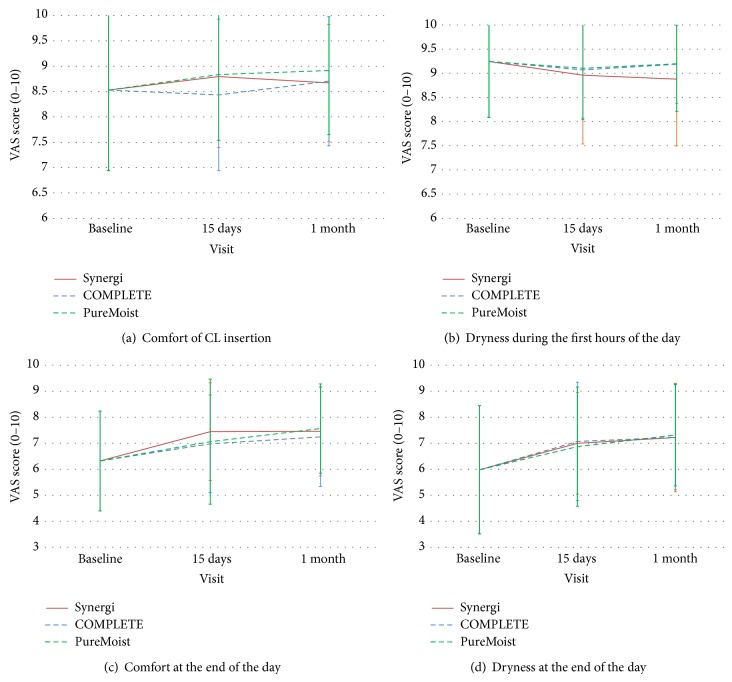
Subjective perception of the subjects regarding comfort upon insertion (a), dryness during first hours of wear (b), end-of-day comfort (c), and end-of-day dryness (d) with the three MPDSs at baseline, 15 days, and 1 month. Error bars represent standard error of mean (SEM).

Comfort at insertion changed from 8.5 with their habitual CL to an average of 8.8 at 1-month visit. End-of-day comfort also increased with the 3 MPDSs from 6.32 with their habitual CL to an average 7.4 at the end of the 1-month period. These results are quite similar to the comfort reported previously by other authors with Biofinity CL [30].  Figure 1 shows the average values of comfort and dryness upon insertion and at the end of the day. Differences between MPDSs for those parameters were not statistically significant at any visit. In the same respect, no statistically significant difference was found for satisfaction neither with the cleanliness nor with the clear vision. Forced choice preference recorded at the end of the study was for Synergi in 26%, COMPLETE RevitaLens in 37%, and OPTI-FREE PureMoist in 37%.

### 3.2. Ocular Surface Signs

No statistically significant differences were observed among the three MPDSs in the slit lamp observations at any visit, except for lid redness and lid roughness. Palpebral conjunctiva showed a statistically significantly different degree of redness between MPDSs on day 30 at the 2-hour visit and a significantly different degree of roughness at the 8-hour visit, as Table 3 shows. To evaluate which MPDSs were different from each other, Mann-Whitney *U* tests were performed. For lid redness at the 2-hour visit, statistically significant differences were seen, being the values lower with COMPLETE RevitaLens than with the other two MPDSs, not existing differences between Synergi and OPTI-FREE PureMoist. Regarding lid roughness, Synergi showed statistically significant higher scores than the other two MPDSs, without observing differences between COMPLETE RevitaLens and OPTI-FREE PureMoist.

**Figure 2:**
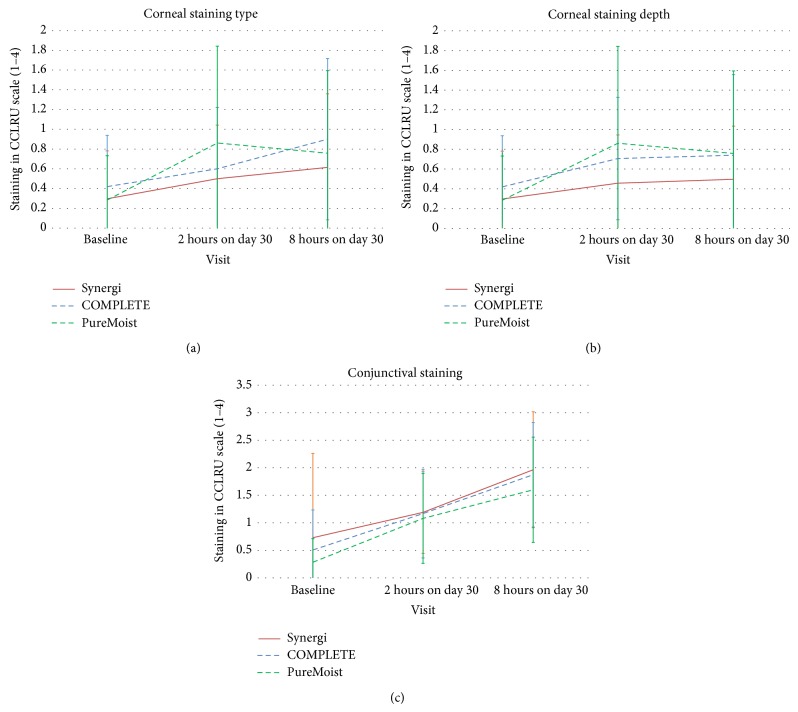
Corneal staining type (a) and depth (b) and conjunctival staining (c) at baseline, 2-hour visit on day 30, and 8-hour visit on day 30. Error bars represent standard error of mean (SEM).

Corneal staining increased with three MPDSs, as Figure 2 shows. Statistically significant changes among visits were found with COMPLETE RevitaLens and OPTI-FREE PureMoist solutions (*P* < 0.05, Kruskal-Wallis test). After analyzing the changes from visit to visit with the Bonferroni test, it was seen that COMPLETE RevitaLens and OPTI-FREE PureMoist increased statistically significantly from baseline to day 30 after 2 hours of lens wear, while Synergi solution increased statistically significantly from baseline to day 30 after 8 hours of CL wear. Conjunctival staining showed a statistically significant increase between each visit with the 3 MPDSs (*P* < 0.05, Bonferroni test). These changes showed an average increase in corneal staining from 0.4 to 0.8 and in conjunctival staining from 0.5 to 1.7, being the staining scores below 3, which is considered as “moderate staining” in the CCLRU scales. SICS pattern was not observed in this study.

**Figure 3:**
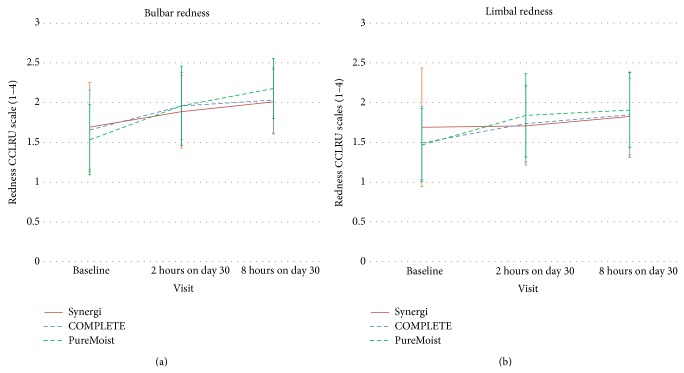
Bulbar redness (a) and limbal redness (b) with each MPDS at baseline, 2-hour visit on day 30, and 8-hour visit on day 30. Error bars represent standard error of mean (SEM).

Bulbar redness showed statistically significant changes with the three MPDSs (*P* < 0.05, Kruskal-Wallis), being the increase statistically significant from baseline to 1-month visit after 2 hours of lens wear for COMPLETE RevitaLens and OPTI-FREE PureMoist (*P* < 0.05, Bonferroni test), remaining stable for the rest of the day with COMPLETE RevitaLens (*P* > 0.05, Bonferroni test). With Synergi and OPTI-FREE PureMoist, a statistically significant increase was seen from 2 to 8 hours of lens wear at 1-month visit (*P* < 0.05, Bonferroni test). Limbal redness showed a significant increase between each visit with COMPLETE RevitaLens and OPTI-FREE PureMoist (*P* < 0.05, Bonferroni test). In general, bulbar redness showed an average change from 1.6 at baseline to 2.1 on day 30 after 8 hours wearing the CLs. Limbal redness showed an average change from 1.5 at baseline to 1.9 on day 30 after 8 hours wearing the CLs. None of these parameters reached the level 3 considered as “moderate redness” in the CCLRU scale, as it is shown in Figure 3.

### 3.3. Precorneal and Prelens Tear Film Parameters

**Figure 4:**
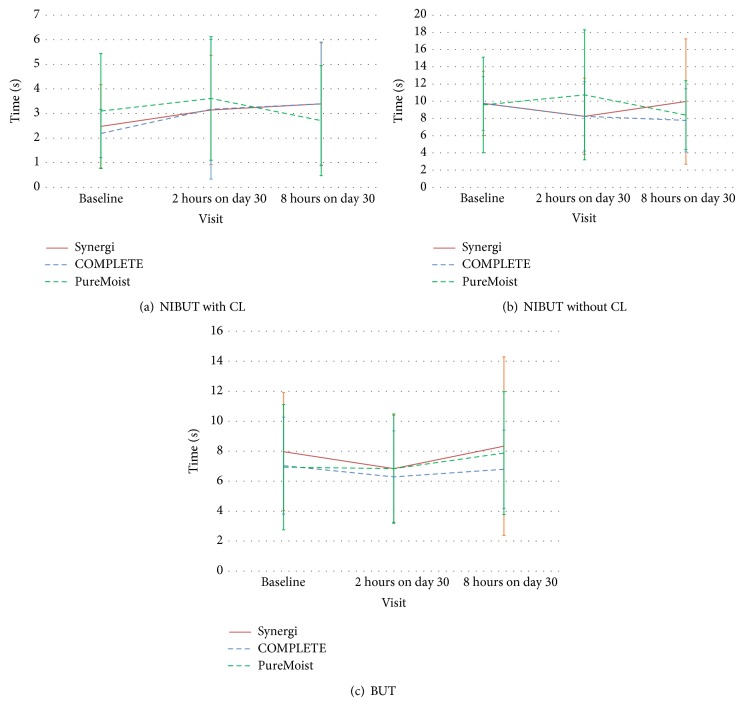
Prelens noninvasive tear break-up time (a), precorneal noninvasive tear break-up time (b), and break-up time without contact lens (c) at baseline, 2-hour visit on day 30, and 8-hour visit on day 30. Error bars represent standard error of mean (SEM).

Precorneal tear film stability (BUT) showed a slight but non-statistically significant decrease at 2-hour visit on day 30, but the values recovered to baseline at 8-hour visit for the three MPDSs. There were no statistically significant changes in NIBUT with CL, neither from the baseline to 1-month visits nor after 1 month from the 2 hours to the 8 hours with any MPDS. However, NIBUT without CL changed between visits with the three MPDSs. With Synergi solution, NIBUT without CL was reduced at 2-hour visit on day 30 compared to baseline but then, it returned to baseline values. With OPTI-FREE PureMoist solution, a decrease was observed from 2 to 8 hours of visit on day 30. With regard to COMPLETE RevitaLens solution, a decrease was observed at 8-hour visit on day 30 compared to baseline values. However, no statistically significant differences among the three MPDSs were observed in any visit.  Figure 4 shows the changes in these parameters with each MPDS. The differences observed between NIBUT with CL and NIBUT without CL may be related to the fact that the NIBUT with CL is more dependent on the lens material surface than the MPDS used, as it has been suggested in a recent study performed also in the CEORLab [31].

### 3.4. Contact Lens Dehydration

Dehydration rates at the 2-hour visit were −2.6 ± 2.4%, −2.3 ± 2.7%, and −1.3 ± 0.9% for Synergi, COMPLETE RevitaLens, and OPTI-FREE PureMoist, respectively (ANOVA, *P* > 0.05). At the 8-hour visit, these values increased to −4.3 ± 3.0%, −3.7 ± 2.8%, and −2.7 ± 1.2%, respectively (ANOVA, *P* > 0.05). COMPLETE RevitaLens and OPTI-FREE PureMoist presented the lower dehydration change from the 2 to 8 hours of visit on day 30 (−1.5 ± 1.1%).

## 4. Discussion

The three MPDSs performed similarly regarding the subjective perception of the subjects and the clinical signs observed at the ocular surface.

SICS staining was not a frequent observation in this clinical study. Despite that, a low-grade of asymptomatic corneal punctate staining was found in most of the subjects after 2 hours of CL wear and it did not disappear after 8 hours of CL use. Garofalo et al. in 2005 [27] found that toxic corneal staining was most likely to be seen between 2 and 4 hours after CL inserting and then became more difficult to be observed. Moreover, a recent study has reported that 36% of subjects showed some degree of SICS in a 1-month study involving the random fitting of Air Optix or Proclear with a hydrogen peroxide system when they were changed to use a PHMB based MPDS and evaluated 2 hours after lens insertion [32]. Instead of being a transient response, the maintenance of the corneal staining during the day suggests that other factors different from the lens solution itself might be related to the “very slight” staining pattern found in the present study. In this regard, it is important to bear in mind that corneal staining may be due to many different conditions related to CL characteristics or to CL wearer conditions [33]. Corneal staining found is on average very low, either for staining type or depth. However, COMPLETE RevitaLens and OPTI-FREE PureMoist showed quite similar staining patterns, being higher than Synergi. This fact may be related to the interaction between the CL and the solution components, but it is not possible to confirm the cause only with the data of the manufacturers.

**Figure 5:**
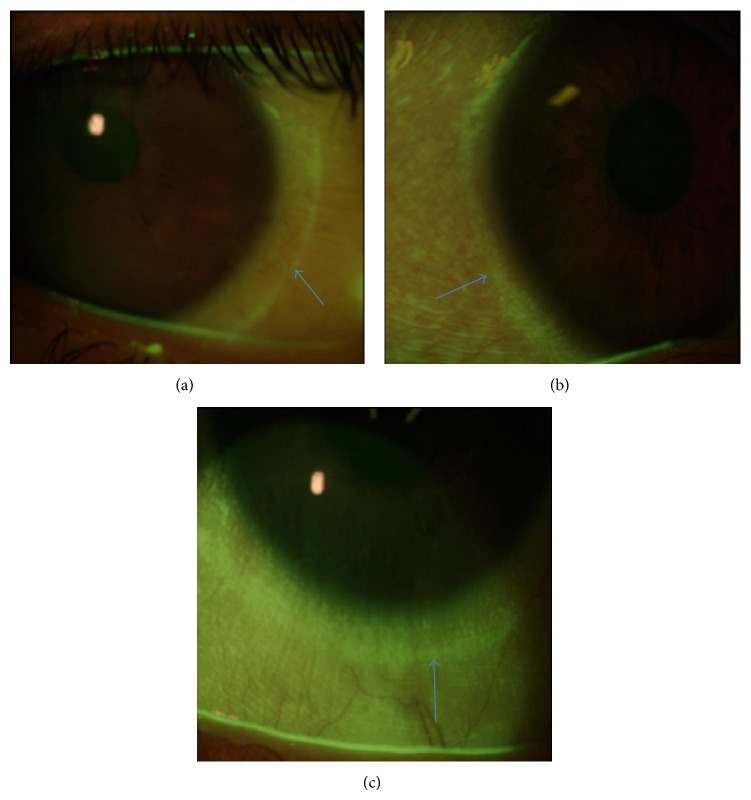
Examples of different degrees of perilimbal conjunctival staining (indicated by the arrows) observed at the 8-hour visit with the three MPDSs. From (a)–(c): from less to more intensive conjunctival staining.

Contrary to the corneal staining, conjunctival staining increased significantly with all MPDSs. A previous study showed a significant increase in conjunctival staining in CL wearers compared to no CL wearers [34]. The most common type of conjunctival staining found was a perilimbal staining, as shown in Figure 5. This result may reflect an ocular response potentially related to CL dehydration or mechanical interaction of the CL with the ocular surface. According to previous studies, conjunctival staining could be related to the lens geometry, especially with the edge lens profile, and the material rigidity [30, 35]. In the present study, all subjects used always the same Si-Hy CL, which suggests that the conjunctival staining may be more related to the CL itself than with the MPDSs used.

The pattern of slight increase in bulbar and limbal redness seems also related to the presence of the CL rather than the MPDS, as a similar pattern was found with the three lens care systems. The changes observed are consistent with those previously reported by other authors [36].

Despite some disputes existing in the literature, several studies have shown similarity in the performance of different MPDS despite pointing to different trends. For instance, some studies have found no differences in comfort [31, 37–40] while others find similar corneal and conjunctival staining [38, 40–43] among different lens care solutions. However, there are also studies that have found differences in comfort [11, 41, 44], corneal staining [11, 25, 44–47], and even in the amount of lens deposits [11, 44, 46].  Table 4 summarizes the main clinical and subjective preference outcomes of different studies comparing the performance of different MPDSs over the past 12 years. Many of these studies focus their attention on comfort and corneal staining. In the present study, the ocular response to three MPDSs that are approved for being used with Si-Hy CLs is shown, evaluating not only the corneal and conjunctival staining but also the bulbar and limbal redness and the tarsal response. The three MPDSs are relatively new in the market and all of them include components to enhance the comfort, surfactants, and wetting agents. In this regard, the comfort with these MPDSs in combination with a Si-Hy CL that is replaced monthly was assed.

Taking into account that lid redness after 2 hours and roughness after 8 hours of CL wear, which could be considered as short and medium term, showed statistically significant differences among the three MPDSs, tarsal response should be also considered in the context of evaluation of the clinical performance of MPDSs at the ocular surface. The tarsal conjunctival tissue is widely recognized for responding to immunological insult. Considering the complex composition of current MPDSs, such response can be used as a relevant biomarker in the evaluation of the clinical performance of modern MPDSs. Contrary to corneal staining and conjunctival staining, or even bulbar redness, lid response has not been evaluated frequently when assessing the ocular response to care solutions [26, 27, 48]. Despite the fact that no significant increase in lid redness or lid roughness was seen with the use of any of the three MPDSs, the lid redness at 2-hour visit and the lid roughness with light reflex at 8-hour visit were statistically significantly. Lid redness was lower with the COMPLETE RevitaLens compared to the other two MPDSs and lid roughness was higher with Synergi compared to the other two MPDSs. This fact may be related to the solution delivery among the day being the lid redness a short response and the lid roughness a more retarded response. In fact, other authors have seen that the lid roughness is reduced with the peroxide use, CL solution without preservatives [49, 50]. Moreover, the components used to keep the surface of the CLs smoother may have some effect on the lid response. Hence, the scientific community should pay more attention to this immunological active location at the ocular surface as it might be a very selective target for some ingredients of the CL care systems.

This study has, however, some important limitations. One of them is that the participants only were evaluated after 1 month using each MPDS. It could be possible that more ocular surface responses take place during the first days after changing the solution. Moreover, the fact that the subjects were not masked about the MPDS may influence the subjective response that was not taken into account.

In summary, the three MPDSs performed similarly regarding the subjective perception of the subjects and the clinical signs observed at the ocular surface. Tarsal conjunctival response should be also considered in the context of the clinical performance of MPDSs at the ocular surface.

## Figures and Tables

**Table 1:** Inclusion and exclusion criteria of the present study.

Inclusion criteria	Exclusion criteria
18 to 35 years of age Absence of ocular disease including dry eye Absence of dry eye symptoms Flat keratometry between 7.60 and 8.10 mm Refractive sphere between −1.00 and −6.00 D Able to understand and sign Consent Form and attend scheduled visits Successful soft contact lens wearers for at least 6 months	Not able to attend visits Symptomatic contact lens wearer Known intolerance to MPDS solutions Dry eye symptoms or signs in the ocular surface (Schirmer <5 mm/5 minutes with closed eyes, corneal or conjunctival staining >grade II in CCLRU grading scales) Taking topical or systemic medication Astigmatism above −0.75 D Other clinically significant ocular findings compatible with inflammation or any other eye condition

**Table 2:** Composition of the MPDS used in the study.

	OPTI-FREE PureMoist	COMPLETE RevitaLens	Synergi
Manufacturer	ALCON	ABBOTT	Sauflon

Disinfecting agent	Polyquaternium-1 0.001% MAPD (ALDOX) 0.0006%	Polyquaternium-1 0.0003% Alexidine 0.00016%	Oxipol Oxychlorite complex (sodium chlorite and hydrogen peroxide)

Buffer	Boric acid; sorbitol	Boric acid, sodium borate decahydrate, sodium chloride; trisodium citrate dehydrate.	Phosphate

Chelating agent	Citrate EDTA 0.05%	EDTA	Not known

Surfactant	Poloxamine (Tetronic 1304)	Tetronic 904	Poloxamer

Wetting agent	HydraGlyde (EOBO-41; polyoxyethylene-poloxybutylene)		Hydroxypropyl methylcellulose (HPMC) Polyvinylpyrrolidone (PVP)

Others	Aminomethyl propanol (AMP-95)		Antimicrobial case

**Table 3:** Values of lid redness and roughness with each MPDS in each visit, showing statistically significant differences in bold.

		Baseline	2 hours on day 30	8 hours on day 30
Lid redness	Synergi	1.40 ± 0.37	**1.62 ± 0.38**	1.65 ± 0.47
	COMPLETE RevitaLens	1.50 ± 0.43	**1.42 ± 0.38**	1.52 ± 0.43
	OPTI-FREE PureMoist	1.49 ± 0.42	**1.66 ± 0.37**	1.58 ± 0.44
	*P*	0.756^∗^	0.044^∗^	0.545^∗^

Lid roughness whit light reflex	Synergi	1.17 ± 0.50	1.23 ± 0.55	**1.45 ± 0.55**
	COMPLETE RevitaLens	1.03 ± 0.59	1.01 ± 0.56	**1.07 ± 0.59**
	OPTI-FREE PureMoist	1.09 ± 0.67	1.04 ± 0.56	**1.13 ± 0.58**
	*P*	0.720^∗^	0.238^∗^	0.026^∗^

^∗^Kruskal-Wallis test.

**Table 4:** Summarized overview of different studies comparing MPDS.

Study	Sample size	Materials	MPDSs	Outcome measures^∗^	Main conclusions
Duench et al. (2013) [47]	15 CL 10 controls	Purevision	ReNu Fresh OPTI-FREE Express	Fluorophotometry	No differences
				Corneal staining	Higher staining with ReNu

De La Jara et al. (2013) [37]	252 subjects	Acuvue Oasys	AQuify ReNu Multiplus OPTI-FREE Express OPTI-FREE Replenish	Comfort at insertion	No differences
				End-of-day comfort End-of-day dryness	Higher comfort and less dryness with MPDSs that contain PHMB than that contain Polyquad
				SICS	No differences between PHMB and Polyquad

González-Méijome et al. (2013) [31]	25 subjects	Air Optix Aqua	COMPLETE RevitaLens Biotrue	NIBUT	No differences
				Comfort	No differences

Campbell et al. (2012) [11]	573 subjects	Not specified	PureMoist ReNu Fresh	Comfort	Better with PureMoist
				Corneal staining	Lower with PureMoist
				Deposits	Less with PureMoist

Martin et al. (2011) [38]	54 subjects	Air Optix	Solo-care Aqua Hidro Health	Corneal edema Endothelial regularity, corneal staining, neovascularization and infiltrates, limbal/bulbar injection, tarsal abnormalities	No differences
				Subjective comfort, average daily wearing time, lens centration, lens deposits, wettability	No differences

Keir et al. (2010) [41]	26 subjects	Air Optix Acuvue OASYS	AOSept Plus OPTI-FREE Replenish	Corneal and conjunctival staining	No differences
				Comfort	AOSept Plus longer comfortable wearing time than OPTI-FREE Replenish

Santodomingo-Rubido et al. (2008) [43]	18 subjects	Premio Acuvue Oasys	MeniCare Soft Complete OPTI-FREE Express	Corneal staining	No differences between MPDSs

Brautaset et al. (2008) [42]	338 subjects	Focus N&D Focus Dailies Acuvue 2 Air Optix Acuvue Advance Freshlook Acuvue Oasys *and others *	OPTI-FREE (Express & Replenish) ReNu Multiplus Complete Moisture Plus Clear Care *and others *	Corneal and conjunctival staining	Conjunctival staining more frequent than corneal staining No differences between CLs or MPDSs

Andrasko and Ryen (2008) [45]	30 subjects^∗∗^	Acuvue 2 Proclear SofLens 66 PureVision Acuvue Advance Acuvue Oasys O2Optix Night & Day Biofinity	Unisol 4 Saline Clear Care OPTI-FREE Express OPTI-FREE Replenish ReNu with Moistureloc^∗∗^ ReNu Multiplus Wal-Mart MPS Target MPS Complete MoisturePLUS AQuify	Corneal staining	OPTI-FREE Express, OPTI-FREE Replenish, and Clear Care exhibited minimal corneal staining area.

Zigler et al. (2007) [39]	233 subjects	Focus N&D Acuvue Advance	OPTI-FREE Replenish ReNu Multiplus Complete MoisturePLUS	Comfort at day 30 Corneal staining Rewetting drop use	No diff. between MPDS Higher staining with ReNu Higher with complete

Stiegemeier et al. (2006) [46]	362 subjects	Acuvue 2 Biomedics 55 Focus 1-2 weeks Frequency 55 Toric Acuvue Biomedics Toric Focus Toric Others	OPTI-FREE Replenish ReNu Multiplus	Comfort Corneal staining Deposits	Higher comfort with OPTI-FREE Replenish compared to ReNu Multiplus Lower corneal staining with OPTI-FREE Replenish Less deposits with OPTI-FREE Replenish

Stiegemeier et al. (2004) [44]	231 subjects	SofLens Surevue	OPTI-FREE Express ReNu Multiplus	Comfort and satisfaction	Higher comfort and satisfaction with OPTI-FREE Express compared to ReNu Multiplus
				Corneal staining	Higher corneal staining with SofLens and ReNu combinations
				Deposits	OPTI-FREE Express tended to be better at maintaining lens cleanliness compared to ReNu Multiplus

Pritchard et al. (2003) [25]	24 subjects	SofLens 66 (alphafilcon A)	OPTI-FREE Express ReNu Multi-Purpose Solution ReNu Multiplus	Corneal staining	Less staining area with OPTI-FREE Express

^∗^Only measures of interest for the purpose of the present study are reported.

^∗∗^ReNu Moistureloc not assayed with all brands due to withdrawal of the product before the end of the study.
